# Supplementary material for: Table-top all-attosecond transient absorption spectroscopy
Source: Nat Commun. 2026 Jul 28;17:7572. doi: 10.1038/s41467-026-76019-z (PMC13415544; doi:10.1038/s41467-026-76019-z)
Supplement: Supplementary file 1 — Supplementary Information [file 41467_2026_76019_MOESM1_ESM.pdf]

Supplementary Information for:

Tabletop All-Attosecond Transient Absorption Spectroscopy

Mikhail Volkov†, Evaldas Svirplys†, Stefanos Carlström†, Serguei Patchkovskii, Misha Yu. Ivanov, Marc J. J. Vrakking, Bernd Schütte*

Max-Born-Institut, Max-Born-Straße 2A, 12489 Berlin, Germany

†These authors contributed equally to this work.

*Corresponding author. Email: Bernd.Schuette@mbi-berlin.de

Contents

1 Experimental Methods . . . 3

2 Investigation of the XUV Spatial Chirp . . . 4

3 Estimation of the NIR Intensity on the Target . . . 4

4 Evaluation of Zero-Time Delay . . . 5

5 AATAS Results in Kr . . . 6

6 Data Analysis . . . 7

7 Rate Equation Calculations . . . 7

8 Spectral Assignments . . . 9

9 Derivation of Single-Atom Response using Perturbation Theory . . . 10

10 Estimating Cationic Coherences . . . 12

11 Energy Structure of Cation . . . 13

11.1 Dirac B-spline R-matrix Calculations . . . 13

11.2 GAMESS-US Calculations . . . 14

11.3 Essential States Model, Energies & Dipole Moments . . . 15

12 Hole Density motion in Ne^+ . . . 17

Supplementary Note 1. Experimental Methods

Supplementary Figs. 1–3 provide additional characterization of the experimental conditions used in this work, including the XUV focal spot sizes (Supplementary Fig. 1), gas-jet density profile and pressure calibration (Supplementary Fig. 2), and representative XUV spectra (Supplementary Fig. 3). The corresponding measurement procedures and analysis methods are described in detail in the Methods section of the main text.

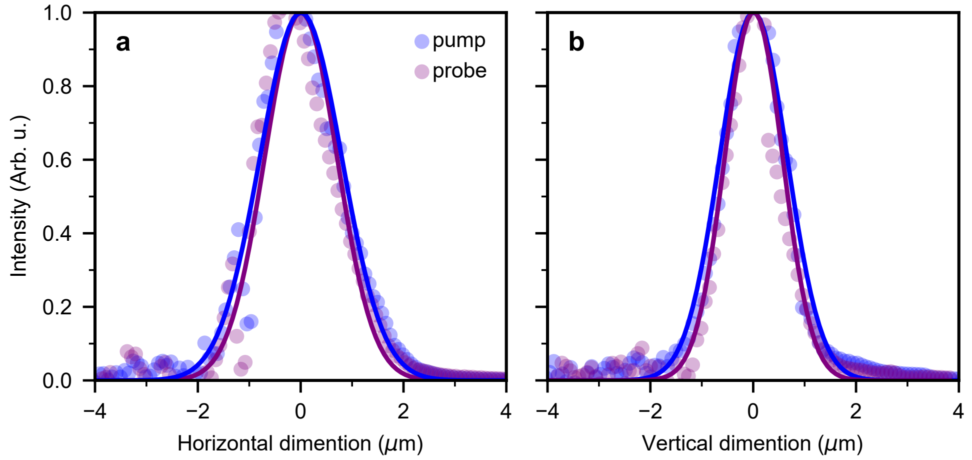

Supplementary Fig. 1 | XUV focus size measurements. a, Measured spatial profiles of the XUV pump (blue) and probe (purple) beams at the focal plane using a knife-edge scan in the horizontal direction. **b**, Same as **a** in the vertical direction. The solid lines show Gaussian fits.

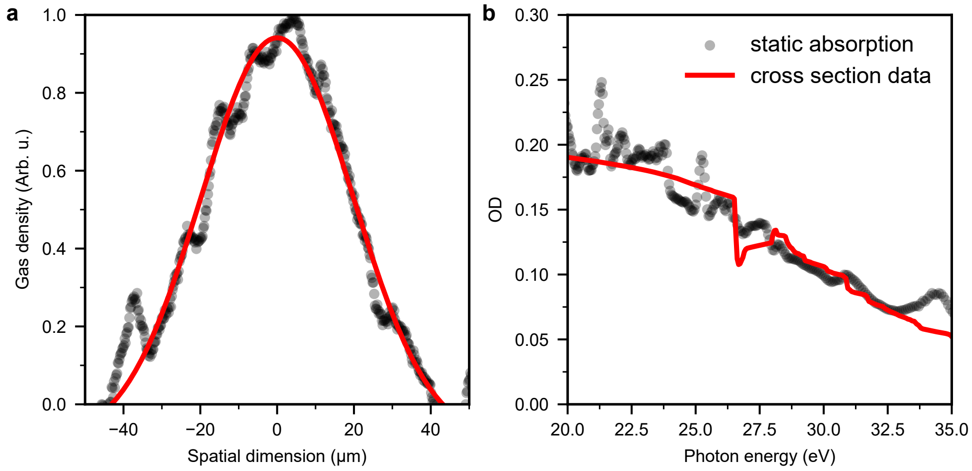

Supplementary Fig. 2 | Gas jet profile and pressure. a, Measured gas jet density across the spatial dimension (black circles) and Gaussian fit (red solid line), giving a FWHM of 46 μm. **b**, Static optical density (OD) measured in Ar (black circles), which is compared with the OD obtained from photoabsorption cross section data [1] (red solid line). The best agreement is obtained for an average pressure of 85 mbar. The resonant features below 26 eV in the experimental data can be attributed to absorption in Ar^+.

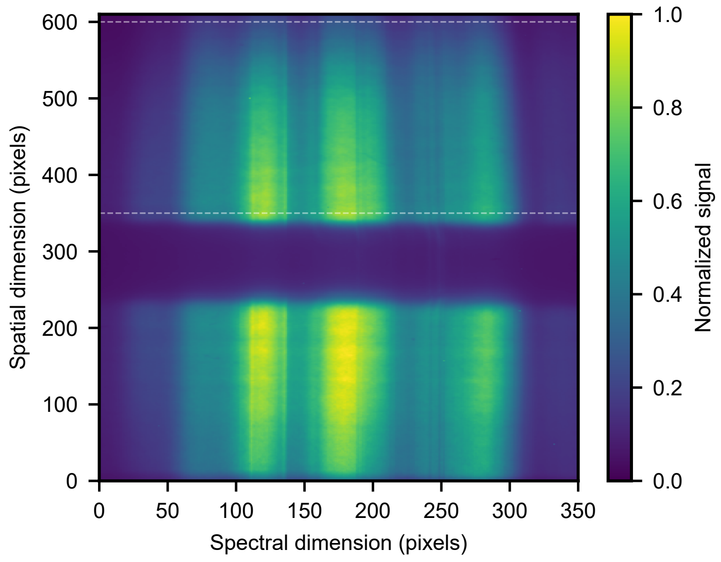

Supplementary Fig. 3 | XUV spectra. The pump (bottom) and the probe (top) beams as they appear on the detector. The gap in the middle corresponds to the gap between the half-cut mirrors in the split-and-delay unit. The dashed lines indicate the spatial region used for the data analysis.

Supplementary Note 2. Investigation of the XUV Spatial Chirp

When focusing XUV beams obtained via HHG, significant chromatic aberration has been observed in a number of studies [2, 3]. To understand whether chromatic aberration is important under our experimental conditions, we have measured and evaluated XUV focus sizes for different harmonics using knife-edge scans (see Supplementary Fig. 4). The results show that the dependence of both the focus size on the harmonic order is small. This observation can be rationalized by taking into account the reshaping of the NIR driving laser pulses in the HHG medium. This is expected to lead to a flat-top driving laser beam profile, which is effectively used for the generation of attosecond pulses [4]. It was recently shown in a theoretical study that this effect may lead to the elimination of chromatic aberration [5].

Supplementary Note 3. Estimation of the NIR Intensity on the Target

While the Al filter used in the experiment attenuates the NIR pulses by several orders of magnitude, it does not completely block it. It is therefore important to estimate the NIR intensity. To this end, we have measured the NIR pulse energy on target, E, giving a value of 60 pJ. Furthermore, the NIR beam waist radii in horizontal and vertical dimensions, $w_{0\mathrm{h}}$ and $w_{0\mathrm{v}}$, were measured as 9.5 µm and 21 µm via knife-edge scans. Taking into account the NIR pulse duration of 3.7 fs, the peak intensity was calculated as $5 \times 10^9\,\mathrm{W/cm^2}$. This is several orders of magnitude smaller than the XUV intensity. The corresponding ponderomotive potential is 0.4 meV, showing that NIR-induced

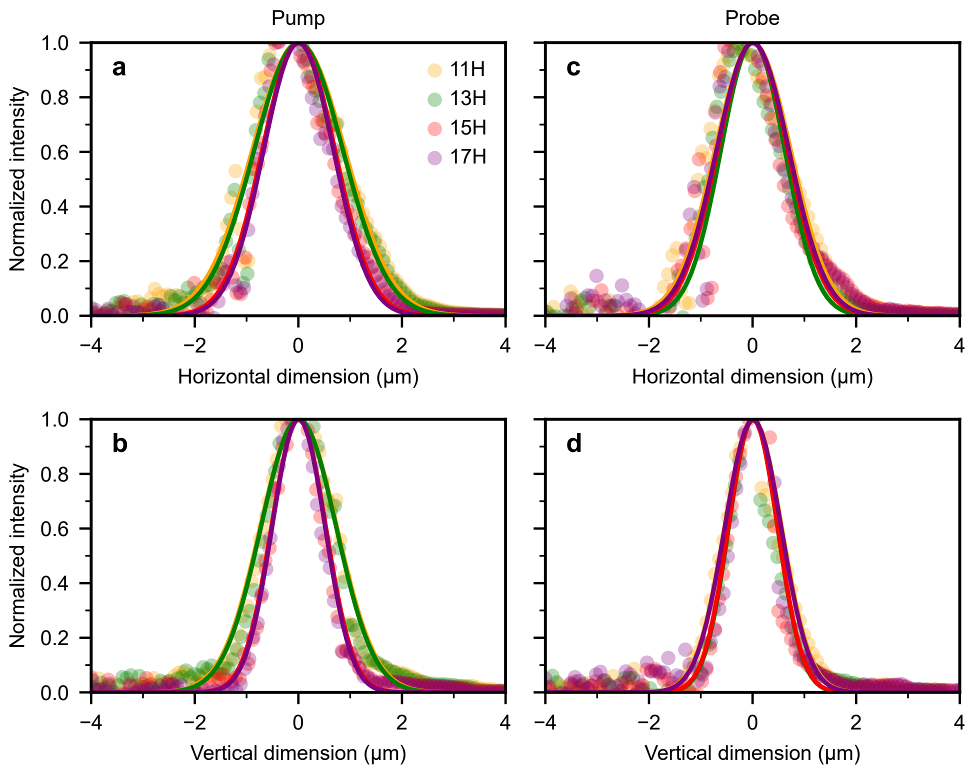

Supplementary Fig. 4 | Spectrally resolved XUV focal profiles. a–b, Spectrally resolved XUV pump focal profiles in the horizontal and vertical directions for the 11th (yellow circles), 13th (green circles), 15th (red circles), and 17th (purple circles) harmonics. **c–d**, same as **a–b**, but for the probe beam. The solid lines are Gaussian fits.

effects are negligible in our experiments.

Supplementary Note 4. Evaluation of Zero-Time Delay

An attractive feature of the presented AATAS scheme is that the assignment of the pump and probe pulses can be reversed. When the pulse usually assigned as the probe pulse serves as the pump pulse and vice versa, pump-induced processes are observed at negative time delays, see Supplementary Fig. 5**b**. When the two delay-dependent signals are normalized, the dynamics are symmetric with respect to time zero. The crossing point of the two curves was used to determine zero-time delay. Importantly, this does not require any information about the underlying physics. This represents an advantage when compared to traditional ATAS experiments using XUV + NIR pulses, where determination of time zero can be difficult.

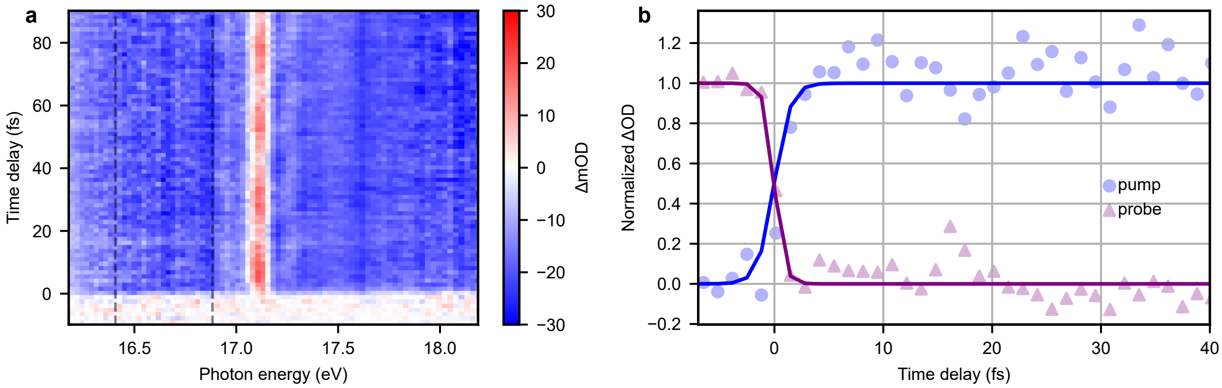

Supplementary Fig. 5 | Evaluation of zero time delay. a, AATAS map in Ar at low photon energies. **b**, normalized transient absorption signal analyzed for the pump and the probe pulse spectra in the photon energy interval from 16.4 to 16.9 eV (circles and triangles). The data were fitted by step functions (solid lines). Zero time delay is determined from the crossing point of the two curves.

Supplementary Note 5. AATAS Results in Kr

In addition to the results presented in the main manuscript, AATAS was performed in Kr, as shown in Supplementary Fig. 6. Oscillations with a period of 6.2 ± 0.2 fs are observed, which is consistent with the spin-orbit splitting of 0.67 eV in Kr^+.

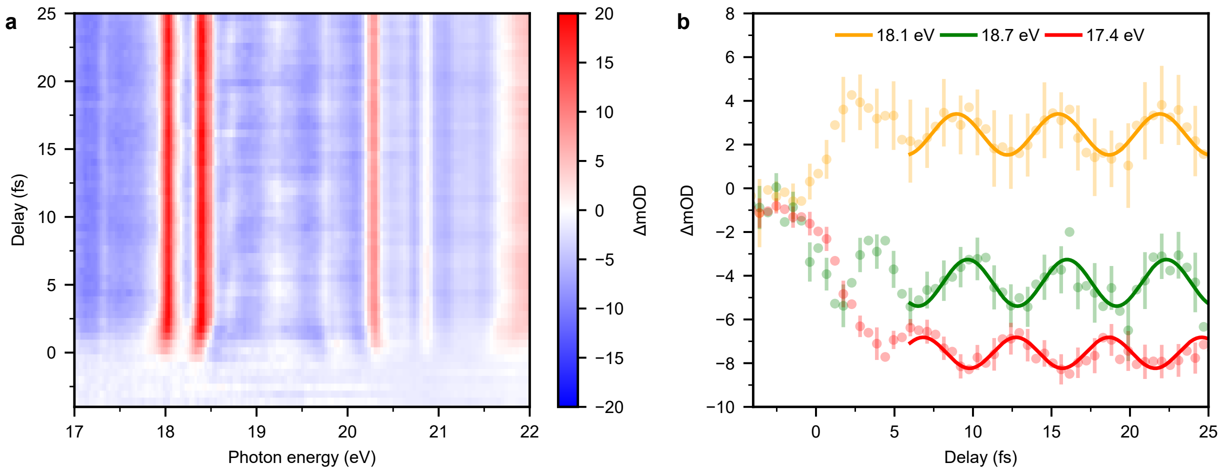

Supplementary Fig. 6 | AATAS results in Kr. a, Differential optical density in Kr as a function of the photon energy and the time delay. **b**, Lineouts at three different photon energies. The solid curves show sinusoidal fits, giving an oscillation period of 6.2 ± 0.2 fs, corresponding to the spin-orbit splitting of 0.67 eV in Kr^+.

Supplementary Note 6. Data Analysis

The transient absorption spectra were analyzed as follows. The acquired XUV spectra for each pump-probe delay were integrated over the selected spatial region as indicated by the dashed lines in Supplementary Fig. 3. The change in the optical density (ΔOD) as a function of the pump-probe delay, τ, was calculated as

$$\Delta\mathrm{OD}(\tau) = -\log_{10}\left(\frac{S(\tau)}{S_0}\right), \tag{1}$$

where $S(\tau)$ is the delay-dependent and spatially integrated probe spectrum, and S_0 is the reference spectrum averaged from data obtained at the first few negative time delays, meaning that the probe pulse arrives before the pump pulse. Spectral calibration was done by measuring absorption peaks in neutral helium and neon and identifying their known spectral resonances [6, 7]. The AATAS data in Xe and Kr were recorded over 20 repeated temporal pump-probe delay scans, with 4,000 laser shots at each delay, while results in Ar and Ne were obtained from 200 consecutive temporal scans with 400 laser shots at each delay. Error bars in all figures represent the standard error of the mean, calculated by determining the standard deviation of ΔOD for each time delay across all measurements and dividing it by the square root of the number of temporal scans recorded.

Supplementary Note 7. Rate Equation Calculations

We have performed rate equation calculations to evaluate the optimal photon fluence in AATAS experiments. We start by considering single-photon ionization, for which, in a perturbative regime, the ionization rate W can be expressed as

$$W = \sigma\frac{\Phi}{\tau}, \tag{2}$$

where σ is the photon energy-dependent cross-section of the species under consideration, Φ is the photon fluence of the XUV pulse, and τ describes the duration of a square-shaped pulse. Assuming the same ionization rate for neutrals and ions, and by solving coupled rate equations, it can be shown that the fraction of singly ionized species after the pulse is

$$f_1 = W\tau e^{-W\tau}. \tag{3}$$

From Eq. (3) it follows that the fraction of singly-ionized species will reach its maximum population when the ionization rate is $W_0 = 1/\tau$. Using Eq. (2), we can rewrite W_0 in terms of the saturation fluence, $\Phi_{sat} = 1/\sigma$, providing an estimate for the optimal fluence that maximizes the ion fraction f_1. Supplementary Fig. 7 shows the calculated ionic fractions for argon, assuming a photon energy of 24 eV. The fractional population of ionized species is plotted as a function of the normalized

fluence Φ/Φ_{sat}. A maximum fraction of 37 % of singly-ionized species is obtained at the saturation intensity. In comparison, the fraction of singly-ionized species is 36 % for the XUV pump fluence used in our experiment.

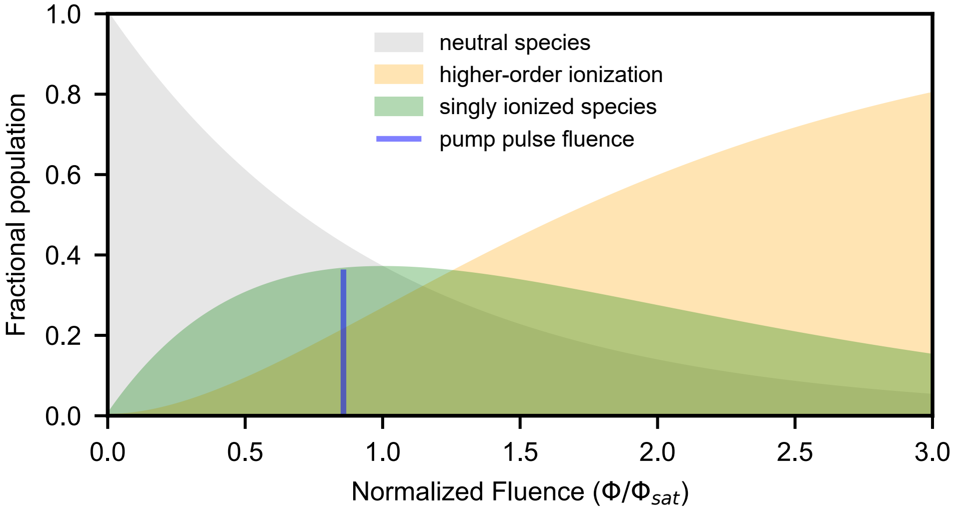

Supplementary Fig. 7 | Calculated fractional populations of neutral and ionized species in AATAS as a function of XUV fluence. Shaded regions represent the relative abundance of neutral atoms (grey area), singly ionized (green area), and higher-order ionized species (yellow area) calculated for argon (cross section of 34.75 Mb [8]) and a photon energy of 24 eV. Vertical line indicate the normalized XUV pump fluence.

Supplementary Note 8. Spectral Assignments

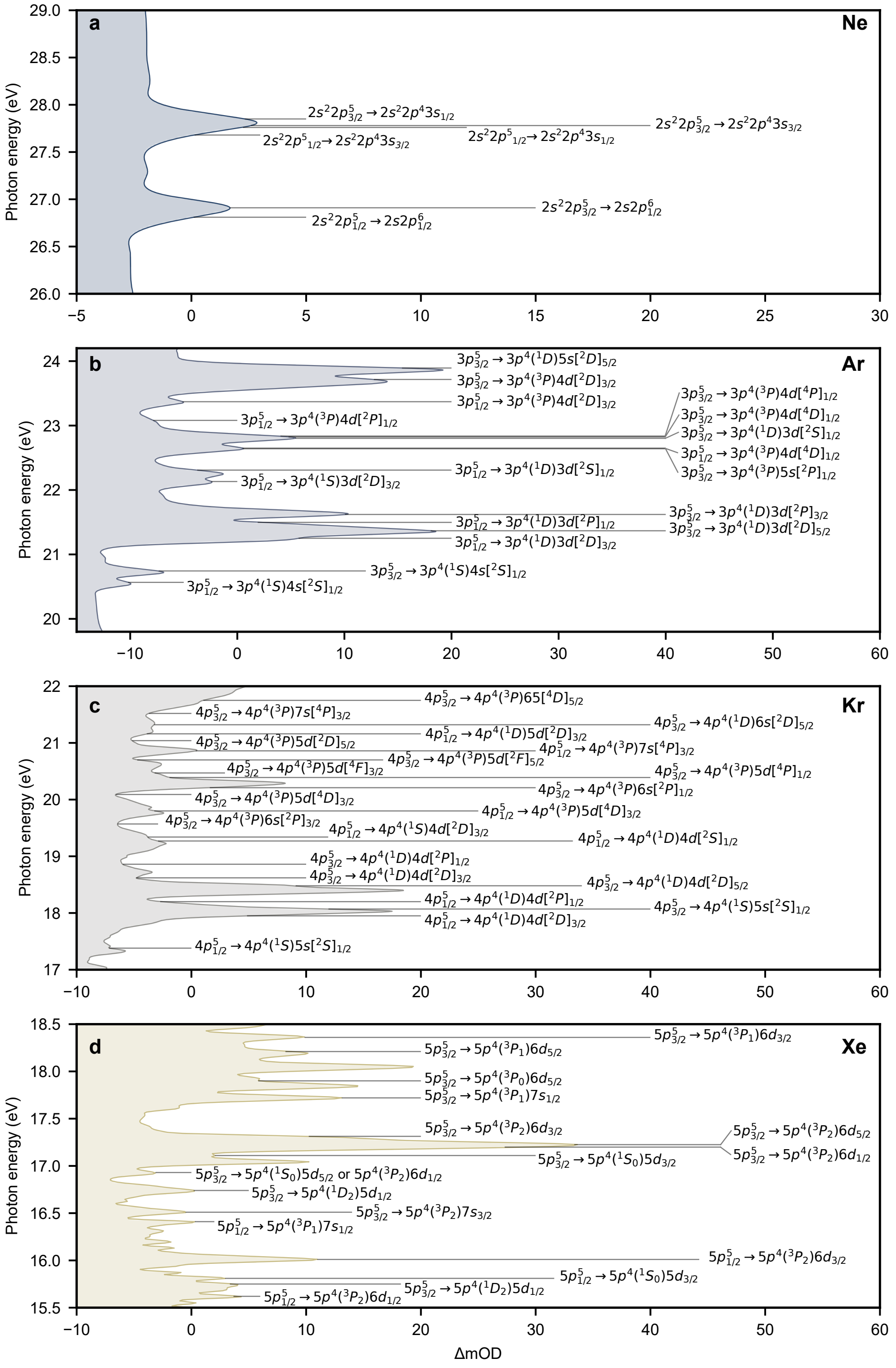

Supplementary Fig. 8 | Spectral assignments of the measured AATAS data. Time-resolved ΔOD maps recorded for all investigated species—(**a**) Ne, (**b**) Ar, (**c**) Kr, and (**d**) Xe—were averaged over positive probe delays to obtain one-dimensional absorption spectra. Spectral line positions were assigned using data from Ref. [9].

Supplementary Note 9. Derivation of Single-Atom Response using Perturbation Theory

We derive a perturbation-theory expression for single-atom response to transient absorption, assuming the pump pulse has via ionization prepared a coherent superposition of the sublevels of the ionic spin-orbit split ground state. The photoelectron leaves the system and is traced out of the density matrix. The ionic coherence is probed by a second pulse that can excite states of the cation. In this section, we use a variation of Einstein's summation convention, where indices only appearing on one side of the equality sign are automatically contracted. Additionally, we use Hartree atomic units, where $\hbar = e = m_e = 4\pi\epsilon = 1$, and the charge of the electron is -1.

The single-atom response is computed as in Ref. [10]

$$S(\omega, t_D) = 2\,\mathrm{Im}\{\tilde{d}(\omega)\tilde{F}^*(\omega)\}, \tag{4}$$

where t_D is the pump–probe pulse time delay. Below, we explore the impact of various initial states.

The dipole spectrum is computed as the Fourier transform of the induced dipole moment:

$$\tilde{d}(\omega) = \frac{1}{\sqrt{2\pi}} \int \mathrm{d}t\; \mathrm{e}^{\mathrm{i}\omega t} d(t), \tag{5}$$

where

$$d(t) = \langle \hat{d} \rangle = \langle \Psi(t) | \hat{d} | \Psi(t) \rangle\,, \tag{6}$$

and the dipole operator for linear polarization is $\hat{d} = -\hat{z}$.

At the level of first-order perturbation theory, the induced dipole moment starting from an initial state

$$|\Psi(t_i)\rangle = u_n\,|n\rangle\,, \quad (\hat{H}_0 - E_n)\,|n\rangle = 0, \tag{7}$$

is given by a sum of two diagrams

$$\tilde{d}^{(1)}(\omega) = -\frac{\mathrm{i}\lambda^1}{\sqrt{2\pi}}$$

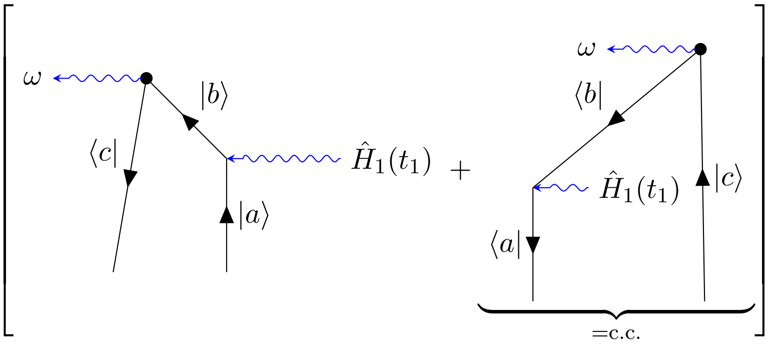

(8)

$$= -\mathrm{i}\lambda^1 \left[\int \mathrm{d}t \int_{t_i}^{t} \mathrm{d}t_1\; u_c^* \mathrm{e}^{\mathrm{i}E_c(t-t_i)} \mathrm{e}^{\mathrm{i}\omega t} d_{cb} \mathrm{e}^{-\mathrm{i}E_b(t-t_1)} F(t_1) d_{ba} \mathrm{e}^{-\mathrm{i}E_a(t_1-t_i)} u_a + \mathrm{c.c.} \right],$$

where λ^1 is the perturbation expansion parameter, and we assume that for eigenstates $|b\rangle$ unoccupied in the initial state (7), there is a finite life-time:

$$\mathrm{Im}\{E_b\} = -\frac{\Gamma_b}{2} \stackrel{\mathrm{def}}{=} -\frac{1}{2\tau_b} < 0. \tag{9}$$

Furthermore, we will assume a Gaussian envelope of the probe pulse:

$$F(t) = F_0 \exp\left(-\frac{t^2}{2\sigma^2}\right)\cos(\omega_0 t) = F_0 \exp\left(-wt^2\right)\frac{\mathrm{e}^{\mathrm{i}\omega_0 t} + \mathrm{e}^{-\mathrm{i}\omega_0 t}}{2}, \quad w \stackrel{\mathrm{def}}{=} \frac{1}{2\sigma^2} = \frac{4\ln 2}{t^2_{\mathrm{FWHM}}}. \tag{10}$$

Focusing on the dynamical part, we find

$$\begin{aligned}\hat{d}(\omega) &= -\frac{\mathrm{i}\lambda^1}{\sqrt{2\pi}}\left[u_c^* u_a \mathrm{e}^{-\mathrm{i}E_{ca}t_i} d_{cb} d_{ba} F_0 g(\omega) + \mathrm{c.c.}\right] \\ &\stackrel{\mathrm{def}}{=} -\frac{\mathrm{i}\lambda^1}{\sqrt{2\pi}}\left[\rho_{ac}(t_i) d_{cb} d_{ba} F_0 g(\omega) + \mathrm{c.c.}\right] \\ &\stackrel{\mathrm{def}}{=} p_{abc} g(\omega) + \mathrm{c.c.},\end{aligned} \tag{11}$$

where

$$\begin{aligned}g(\omega) &= \int \mathrm{d}t \int_{t_i}^{t} \mathrm{d}t_1\, \mathrm{e}^{\mathrm{i}E_c t}\mathrm{e}^{\mathrm{i}\omega t}\mathrm{e}^{-\mathrm{i}E_b(t-t_1)}\frac{F(t_1)}{F_0}\mathrm{e}^{-\mathrm{i}E_a t_1} \\ &= \int \mathrm{d}t \int_{t_i}^{t} \mathrm{d}t_1\, \mathrm{e}^{\mathrm{i}E_c t}\mathrm{e}^{\mathrm{i}\omega t}\mathrm{e}^{-\mathrm{i}E_b(t-t_1)}\mathrm{e}^{-wt_1^2}\frac{\mathrm{e}^{\mathrm{i}\omega_0 t_1} + \mathrm{e}^{-\mathrm{i}\omega_0 t_1}}{2}\mathrm{e}^{-\mathrm{i}E_a t_1} \\ &= \int \mathrm{d}t\, \mathrm{e}^{\mathrm{i}(E_{cb}+\omega)t}\int_{t_i}^{t} \mathrm{d}t_1\, \mathrm{e}^{-wt_1^2}\frac{\mathrm{e}^{\mathrm{i}(E_{ba}+\omega_0)t_1} + \mathrm{e}^{\mathrm{i}(E_{ba}-\omega_0)t_1}}{2}\end{aligned} \tag{12}$$

Because of the finite life time imposed on $|b\rangle$ [cf. (9)], $\mathrm{Re}\{\mathrm{i}E_{cb}\} = \mathrm{Re}\{\mathrm{i}(E_c - E_b)\} = \mathrm{Im}\{E_b\} < 0$. $g(\omega)$ is thus essentially the Fourier transform of an exponentially damped oscillating function (i.e. a Lorentz profile, as expected). The inner integral mainly serves to set the initial amplitude of the oscillations; its transient behaviour, which quickly passes, is due to the probe pulse $\mathrm{e}^{-wt_1^2}\cos(\omega_0 t)$, and the long-term behaviour is found by setting the upper limit to $+\infty$. Since we are later going to multiply $g(\omega)$ by $\tilde{F}^*(\omega)$, the lower limit t_i is immaterial as well, and we may set it to $-\infty$. Finally, employing the rotating wave approximation (RWA), inner integral amounts to the Fourier transform of the probe pulse envelope $\mathrm{e}^{-wt_1^2}$ evaluated at $E_{ba} - \omega_0$.

$$\exp\left(-\alpha t^2\right) \leftrightarrow \frac{1}{\sqrt{2\alpha}}\exp\left(-\frac{\omega^2}{4\alpha}\right), \tag{13}$$

assuming symmetric normalization $(2\pi)^{-1/2}$ of the Fourier transform.

$$\Longrightarrow \int_{t_i}^{t} \mathrm{d}t_1\, \mathrm{e}^{-wt_1^2} \frac{\mathrm{e}^{\mathrm{i}(E_{ba}+\omega_0)t_1} + \mathrm{e}^{\mathrm{i}(E_{ba}-\omega_0)t_1}}{2} \approx \underbrace{\frac{1}{2}\sqrt{\frac{\pi}{w}} \exp\left[-\frac{(E_{ba}-\omega_0)^2}{4w}\right]}_{\stackrel{\text{def}}{=} Q_{ba}} \theta(t). \tag{14}$$

$$\begin{aligned} \Longrightarrow\ g(\omega) &\approx Q_{ba} \int \mathrm{d}t\ \mathrm{e}^{\mathrm{i}(E_{cb}+\omega)t}\theta(t) \\ &= Q_{ba}\sqrt{2\pi}\sqrt{\frac{\pi}{2}} \frac{1}{\mathrm{i}\pi(E_{cb}+\omega)+0^+} = -\frac{Q_{ba}}{\mathrm{i}(E_{bc}-\omega)+0^+} \end{aligned} \tag{15}$$

Our final perturbation-theory expression for the single-atom response is

$$\begin{aligned} S(\omega) &= 2\,\mathrm{Im}\{F^*(\omega)\hat{d}(\omega)\} = 2\,\mathrm{Im}\{F^*(\omega)[p_{abc}g(\omega) + \cancel{\mathrm{c.c.}}]\} \\ &\approx 2\,\mathrm{Im}\left\{\frac{F_0}{2}\sqrt{\frac{\pi}{w}} \exp\left[-\frac{(\omega-\omega_0)^2}{4w}\right]\left[-\frac{p_{abc}Q_{ba}}{\mathrm{i}(E_{bc}-\omega)+0^+}\right]\right\} \\ &\stackrel{\text{def}}{=} 2\Phi(\omega)\,\mathrm{Im}\left\{\exp\left[-\frac{(E_{ba}-\omega_0)^2}{4w}\right]\frac{\rho_{ac}(t_i)d_{cb}d_{ba}}{(E_{bc}-\omega)-\mathrm{i}0^+}\right\}, \end{aligned} \tag{16}$$

where the prefactor

$$\Phi(\omega) \stackrel{\text{def}}{=} \frac{\lambda^1}{\sqrt{2\pi}} \frac{F_0^2}{4} \frac{\pi}{w} \exp\left[-\frac{(\omega-\omega_0)^2}{4w}\right] \tag{17}$$

is a fairly smooth function that we may approximate as a constant, provided the states $|a\rangle$, $|b\rangle$, and $|c\rangle$ are within the bandwidth $\sim \sqrt{w}$ of the carrier frequency ω_0.

Supplementary Note 10. Estimating Cationic Coherences

Supplementary Table 1 | Initial coherences between the cationic states of neon, following ionization by a 300 as pulse of $1\,\mathrm{W\,cm^{-2}}$ peak intensity. The cationic states are labelled by the dominant holes relative to the neutral ground states, and the numbers in the parentheses are the m_j quantum numbers of the holes, equal to $-M_J$ of the cationic states. In the table, we use the following values $a = 0.12276$, $b = 0.212508$, $c = 0.164693$, $d = 0.063$, $\theta = 9.2°$, $\phi = 170.8°$.

	$2\mathrm{p}_{3/2}^{-1}(3/2)$	$2\mathrm{p}_{3/2}^{-1}(1/2)$	$2\mathrm{p}_{3/2}^{-1}(-3/2)$	$2\mathrm{p}_{3/2}^{-1}(-1/2)$	$2\mathrm{p}_{1/2}^{-1}(-1/2)$	$2\mathrm{p}_{1/2}^{-1}(1/2)$
$2\mathrm{p}_{3/2}^{-1}(3/2)$	a					
$2\mathrm{p}_{3/2}^{-1}(1/2)$		b				$d\mathrm{e}^{-\mathrm{i}\theta}$
$2\mathrm{p}_{3/2}^{-1}(-3/2)$			a			
$2\mathrm{p}_{3/2}^{-1}(-1/2)$				b	$d\mathrm{e}^{\mathrm{i}\phi}$	
$2\mathrm{p}_{1/2}^{-1}(-1/2)$				$d\mathrm{e}^{-\mathrm{i}\phi}$	c	
$2\mathrm{p}_{1/2}^{-1}(1/2)$		$d\mathrm{e}^{\mathrm{i}\theta}$				c

As we see in Equation (16), we need the initial coherences ρ_{ab}. We compute these using time-dependent configuration-interaction singles calculations [11, 12], by ionizing the neutral neon atom and tracing out the photoelectron from the density matrix. The time-dependent calculations were performed on a finite-differences grid spanning from 0 Bohr to 100 Bohr covered by 577 points with a grid spacing varying in a log-linear fashion:

$$r_j = r_{j-1} + \rho_{\text{min}} + (1 - \mathrm{e}^{-\alpha r_{j-1}})(\rho_{\text{max}} - \rho_{\text{min}}), \quad r_1 = \rho_{\text{min}}/2, \tag{18}$$

where $\rho_{\text{min}} = 0.13\,\text{Bohr}$, $\rho_{\text{max}} = 0.18\,\text{Bohr}$, and $\alpha = 0.1$. In the spin–angular dimension, spinor-spherical harmonics were used, with $\ell \leq 10$. The complex-absorbing potential by Ref. [13] was used to avoid reflections from the end of the computational box. Spin–orbit interaction was accounted for using the effective-core potential of Ref. [14]. Photoelectron spectra were computed using a multichannel extension of the time-dependent surface-flux technique [11, 12, 15].

The ionizing pulse has a Gaussian envelope, and a photon energy centred at $1\,\text{Ha} \approx 27.211\,\text{eV}$. The pulse duration (FWHM) was varied from 150 as to 450 as, which only had a slight impact on the phases of the coherences (about 5° per 150 as). The peak intensity was chosen as $1\,\text{W}\,\text{cm}^{-2}$, $100\,\text{W}\,\text{cm}^{-2}$, and $1000\,\text{W}\,\text{cm}^{-2}$. After normalizing the ionic coherences to the sum of the diagonal of the reduced density matrix (i.e. the total ionization yield), both the relative populations as well as the relative phases proved very stable to the intensity variations, in keeping with the perturbative nature of the process. The results presented in Supplementary Table 1 were computed for 300 as duration and $1\,\text{W}\,\text{cm}^{-2}$ peak intensity. Since the tabulated values are *hole coherences*, the *initial state coherences* needed in Equation (16) are obtained by changing the sign of the coherences, i.e. substituting $(\theta, \phi) \to (-\phi, -\theta)$.

Supplementary Note 11. Energy Structure of Cation

11.1 Dirac B-spline R-matrix Calculations

The energy structure is computed using the DBSR suite of programs by Oleg Zatsarinny [16–20]. The level of theory is the Dirac–Coulomb Hamiltonian, which we solve using Dirac–Hartree–Fock for the reference, and then we add correlation using a multiconfigurational treatment (MCDHF).

Our *Ansatz* is a close-coupling expansion:

$$|\Psi\rangle = \hat{\mathcal{A}}\,|\Phi_j^N\rangle\;|\chi_k\rangle\,, \tag{19}$$

where $|\Phi_j^N\rangle$ is the set of target states (ionization channels), $|\chi_k\rangle$ the set of "free" electrons (it is used to represent both highly excited, but bound, Rydberg states, as well as truly free continuum electrons), and $\hat{\mathcal{A}}$ is the antisymmetrization operator. As is customary in scattering calculations, N is the number of electrons in the scattering target (atom or ion), and the scattering electron is

the "+1".

The only good quantum numbers in the relativistic case are the total angular momentum J of the many-body wavefunction, and the overall parity Π. We use the notation $|\gamma J^{\Pi}\rangle$ for states, where γ is the configuration and any other quantum numbers necessary to disambiguate states. To get good agreement with experimental energies, correlation is very important, and we approximate the states in a multiconfigurational expansion as

$$|\gamma J^{\Pi}\rangle \approx c^{(0)}\,|\gamma J^{\Pi}\rangle^{(0)} + c_i^{(1)}\,|\gamma J^{\Pi}\rangle_i^{(1)} + c_j^{(2)}\,|\gamma J^{\Pi}\rangle_j^{(2)} + ... \tag{20}$$

where $|\gamma J^{\Pi}\rangle_i^{(w)}$ represents a state where we have made w orbital substitutions with respect to the reference state $|\gamma J^{\Pi}\rangle^{(0)}$ (typically the DHF solution). We truncate the expansion at singles and doubles, i.e. $w \leq 2$. We also do not include all possible substitutions, instead we successively add *layers* of correlation orbitals until the calculation is converged; if one layer i contains correlation orbitals that mostly look like $n_i\ell_i$ orbitals, where $\ell_i < n_i$, the next layer will consist of orbitals $n_{i+1}\ell_{i+1}$, where $n_{i+1} = n_i + 1$, $\ell_{i+1} < n_{i+1}$, and so on. In the self-consistent calculations, only the newly added orbitals are optimized, where as all the expansion coefficients $\mathbf{c}$ are recalculated. Typically, state-averaging is performed, where the orbitals are optimized for a specific set of chosen states, but the same set of orbitals are used for all states (which have different expansion coefficients).

The calculations include all partial waves needed to support the highest correlation orbital 3d. The radial functions are expanded in a B-spline basis of polynomial order 8 and 9, for the large and small components, respectively. For the target states used in the close-coupling expansion, the radial grid extends to 20 Bohr. The resulting target states are shown in Supplementary Table 2. The free electron is expanded over the same basis, but extending to 50 Bohr, and the close-coupling Hamiltonian is diagonalized. The reduced dipole matrix elements are computed from the resulting eigenstates, with the angular factors added to form the Cartesian dipole matrix elements. Dipole matrix elements computed using DBSR are not guaranteed to follow a consistent sign convention. We therefore fix the signs using an auxiliary calculation using the molecular GAMESS-US code (see next subsection).

11.2 GAMESS-US Calculations

The dipole-coupling pattern between states of interest was calculated at the DK-SO-MCQDPT2 [21–27] + aug-cc-pCVTZ-DK [28–30] level of theory, in the basis of the ten spatial components of the scalar-relativistic states $1^2\mathrm{P^o}$ ($1^2\mathrm{B_{1u}}$, $1^2\mathrm{B_{2u}}$, and $1^2\mathrm{B_{3u}}$ in the computational D_{2h} point group), $1^2\mathrm{S}$ ($1^2\mathrm{A_g}$ in D_{2h}), $1^4\mathrm{P}$ ($1^4\mathrm{B_{1g}}$, $1^4\mathrm{B_{2g}}$, $1^4\mathrm{B_{3g}}$ in D_{2h}), and $1^2\mathrm{P}$ ($1^2\mathrm{B_{1g}}$, $1^2\mathrm{B_{2g}}$, $1^2\mathrm{B_{3g}}$ in D_{2h} spanning the 7-electron space within the $2\mathrm{s}, 2\mathrm{p}^3, 3\mathrm{s}$ cation manifold [CAS(7,5)]. In the CASSCF orbital optimization, each of the spatial components of the ground $1^2\mathrm{P^o}$ state was included with the weight of $\frac{3}{16}$, while the excited states were weighted with $\frac{1}{16}$ each. The M_J components of

Supplementary Table 2 | Energies of select states of neutral neon (Ne I), the neon cation (Ne II), and the neon dication (Ne III), obtained by MCDHF calculations including correlation orbitals up to 3s − d.

Stage	Configuration	J^{Π}	E [eV]	Exp [eV]	Error [eV]
I	$2s^2\ 2p_{1/2}^2\ 2p_{3/2}^4$	0^e	0.000 000	0.0	0.0
II	$2s^2\ 2p_{1/2}^2\ 2p_{3/2}^3$	$(3/2)^o$	20.891 564	21.5645	−0.672 977
II	$2s^2\ 2p_{1/2}\ 2p_{3/2}^4$	$(1/2)^o$	20.991 083	21.6613	−0.670 219
II	$2s\ 2p_{1/2}^2\ 2p_{3/2}^4$	$(1/2)^e$	48.362 639	48.475	−0.112 374
III	$2s^2\ 2p_{1/2}^2\ 2p_{3/2}^2$	2^e	61.161 603	62.5275	−1.365 91
III	$2s^2\ 2p_{1/2}\ 2p_{3/2}^3$	1^e	61.241 844	62.6072	−1.365 37
III	$2s^2\ 2p_{3/2}^4$	0^e	61.268 315	62.6416	−1.373 33
III	$2s^2\ 2p_{1/2}\ 2p_{3/2}^3$	2^e	64.526 969	65.7314	−1.204 38
III	$2s^2\ 2p_{1/2}^2\ 2p_{3/2}^2$	0^e	69.518 551	69.44	0.078 590 1
III	$2s\ 2p_{1/2}^2\ 2p_{3/2}^3$	2°	86.684 093	87.8562	−1.172 15
III	$2s\ 2p_{1/2}\ 2p_{3/2}^4$	1°	86.761 450	87.9285	−1.167 03
III	$2s\ 2p_{1/2}\ 2p_{3/2}^4$	0°	86.772 591	87.9683	−1.195 73

the spin-orbit states were additionally split for the transition-dipole classification, by applying a perturbation $\hat{H}_{BJ} = B_z(\hat{L}_z + \hat{S}_z)$, with the pseudo-magnetic field $B = 10^{-5}$ atomic units. The final energies of the states are summarized in Supplementary Table 3. All quantum-chemistry calculations were performed using GAMESS-US [31, 32].

Supplementary Table 3 | Calculated and experimental energies of the electronic states, in Hartree. The calculated energies are relative to the total energy of the $^2P^o_{3/2}$ state (−128.225 840 Ha).

State	Excitation	Experiment [33]
$^2P^o_{3/2}$	0.0	0.0
$^2P^o_{1/2}$	0.003 344	0.003 556
$^2S_{1/2}$	0.990 503	0.988 942
$^4P_{5/2}$	0.989 928	0.998 433
$^4P_{3/2}$	0.992 217	1.000 792
$^4P_{1/2}$	0.993 909	1.002 154
$^2P_{3/2}$	1.014 915	1.021 016
$^2P_{1/2}$	1.017 695	1.023 805

11.3 Essential States Model, Energies & Dipole Moments

In the essential states model, we include 26 states, whose designations and experimental energies [33] are given in Supplementary Table 4. Due to an abundance symmetry present in the problem, many of the dipole matrix elements between the essentials states are of equal magnitude. Here, we only

Supplementary Table 4 | Essential states used in the perturbation-theory calculation. For states 7–26, the imaginary part of the energy is $\mathrm{Im}\{E\} = -\Gamma/2$, where the decay rate is chosen as $\Gamma = 9.676 \times 10^{-4}$ Ha $\sim \hbar/25$ fs.

States	Configuration	J level	M_J sublevels	Re$\{E\}$ [Ha]
1–4	$2s^2\ 2p^5\ {}^2P^o$	3/2	$-3/2, -1/2, 1/2, 3/2$	0
5–6		1/2	$-1/2, 1/2$	0.003 555 873
7–8	$2s\ 2p^6\ {}^2S$	1/2	$-1/2, 1/2$	0.988 941 62
9–14	$2s^2\ 2p^4\ 3s\ {}^4P$	5/2	$-5/2, -3/2, -1/2, 1/2, 3/2, 5/2$	0.998 433 211
15–18		3/2	$-3/2, -1/2, 1/2, 3/2$	1.000 791 861
19–20		1/2	$-1/2, 1/2$	1.002 154 299
21–24	$2s^2\ 2p^4\ 3s\ {}^2P$	3/2	$-3/2, -1/2, 1/2, 3/2$	1.021 015 54
25–26		1/2	$-1/2, 1/2$	1.023 805 212

give the non-zero matrix elements appearing above the diagonal. The ones below the diagonal are trivially recovered since the matrix representation of $\hat{z}$ is Hermitian.

- $z_{1,10} = z_{4,13} = -0.002\,713\,072$
- $-z_{1,15} = z_{4,18} = 0.004\,461\,812$
- $-z_{1,21} = +z_{4,24} = 0.289\,085\,735$
- $z_{2,7} = z_{3,8} = 0.301\,304\,860$
- $z_{2,11} = z_{3,12} = -0.003\,322\,821$
- $-z_{2,16} = z_{3,17} = 0.001\,487\,271$
- $z_{2,19} = z_{3,20} = -0.017\,628\,096$
- $-z_{2,22} = z_{3,23} = 0.096\,361\,912$
- $z_{2,25} = z_{3,26} = -0.132\,520\,663$
- $z_{5,7} = -z_{6,8} = 0.204\,296\,894$
- $z_{5,16} = z_{6,17} = 0.003\,697\,899$
- $z_{5,19} = -z_{6,20} = 0.012\,549\,756$
- $z_{5,22} = z_{6,23} = -0.141\,135\,324$
- $z_{5,25} = -z_{6,26} = 0.196\,911\,678$

Supplementary Note 12. Hole Density motion in Ne^+

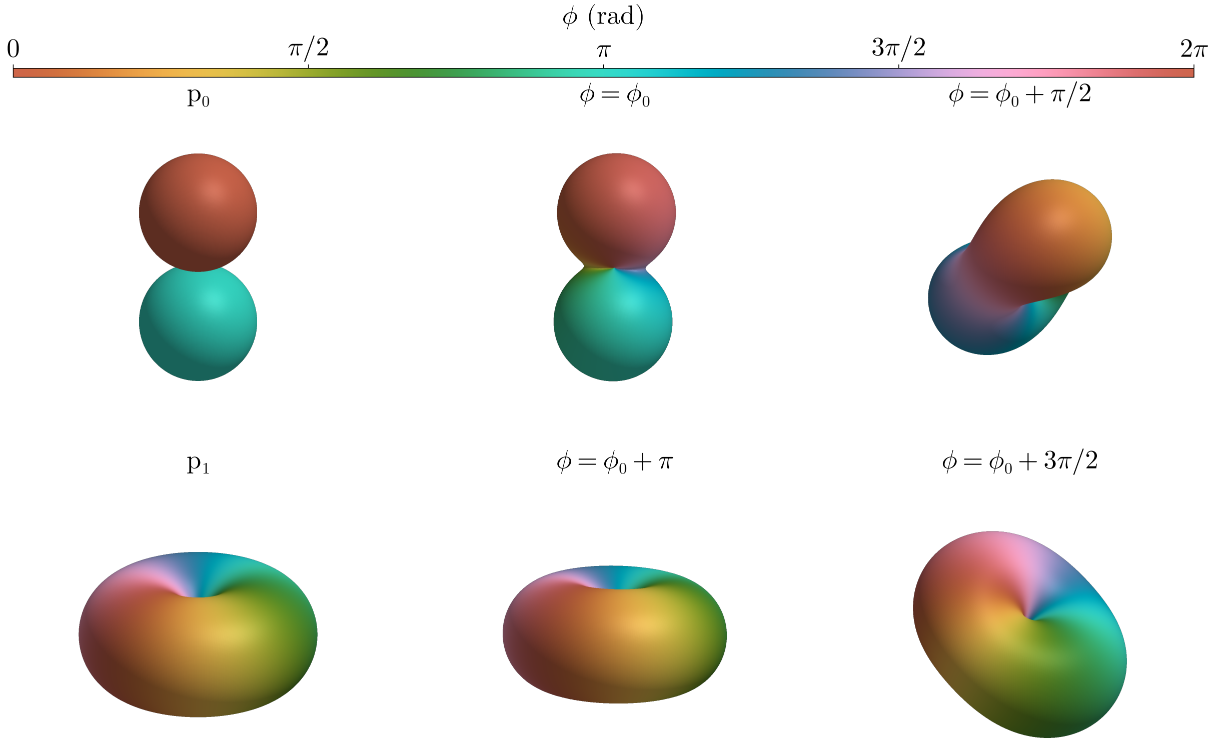

Supplementary Fig. 9 | Time-dependent hole density in the $m_j = 1/2$ channel of neon, for a few different values of the phase $\phi = \Delta E_{\mathbf{so}} t$. ϕ_0 was chosen to maximize the visual overlap with p_0. This happens at $\phi_0 = -0.3$ rad, corresponding to about -2 fs for the spin–orbit splitting of Ne.

Supplementary References

[1] Carlson, R. W., Judge, D. L., Ogawa, M. & Lee, L. C. Photoabsorption Cross Section of Argon in the 180–700-A Wavelength Region. *Applied Optics* **12**, 409_1–412 (1973). URL https://opg.optica.org/ao/abstract.cfm?uri=ao-12-2-409_1. Publisher: Optica Publishing Group.

[2] Quintard, L. *et al.* Optics-less focusing of XUV high-order harmonics. *Science Advances* **5**, eaau7175 (2019). URL https://www.science.org/doi/10.1126/sciadv.aau7175. Publisher: American Association for the Advancement of Science.

[3] Wikmark, H. *et al.* Spatiotemporal coupling of attosecond pulses. *Proceedings of the National Academy of Sciences* **116**, 4779–4787 (2019). URL https://pnas.org/doi/full/10.1073/pnas.1817626116.

[4] Major, B. *et al.* Propagation-assisted generation of intense few-femtosecond high-harmonic pulses. *Journal of Physics: Photonics* **2**, 034002 (2020). URL https://dx.doi.org/10.1088/2515-7647/ab869d. Publisher: IOP Publishing.

[5] Li, B. *et al.* Elimination of chromatic aberration in high-order harmonic generation using a plasma-induced flat-top beam in a gas medium. *Physical Review A* **110**, 043511 (2024). URL https://link.aps.org/doi/10.1103/PhysRevA.110.043511. Publisher: American Physical Society.

[6] Baig, M. A. & Connerade, J. P. Centrifugal barrier effects in the high Rydberg states and autoionising resonances of neon. *Journal of Physics B: Atomic and Molecular Physics* **17**, 1785 (1984). URL https://dx.doi.org/10.1088/0022-3700/17/9/017.

[7] Herzberg, G. Ionization potentials and Lamb shifts of the ground states of 4He and 3He. *Proceedings of the Royal Society of London. Series A. Mathematical and Physical Sciences* **248**, 309–332 (1997). URL https://royalsocietypublishing.org/doi/10.1098/rspa.1958.0246. Publisher: Royal Society.

[8] Chan, W. F., Cooper, G., Guo, X., Burton, G. R. & Brion, C. E. Absolute optical oscillator strengths for the electronic excitation of atoms at high resolution. III. The photoabsorption of argon, krypton, and xenon. *Physical Review A* **46**, 149–171 (1992). URL https://link.aps.org/doi/10.1103/PhysRevA.46.149. Publisher: American Physical Society.

[9] Kramida, A., Ralchenko, Y., Reader, J. & Team, N. A. NIST Atomic Spectra Database (version 5.12) (2024). URL https://physics.nist.gov/asd. Place: Gaithersburg, MD.

[10] Wu, M., Chen, S., Camp, S., Schafer, K. j. & Gaarde, M. B. Theory of strong-field attosecond transient absorption. *Journal of Physics B: Atomic, Molecular and Optical physics* **49**, 062003 (2016). URL http://dx.doi.org/10.1088/0953-4075/49/6/062003. Publisher: IOP Publishing.

[11] Carlström, S., Bertolino, M., Dahlström, J. M. & Patchkovskii, S. General time-dependent configuration-interaction singles. II. Atomic case. *Physical Review A* **106**, 042806 (2022). URL https://arxiv.org/abs/2204.10534. Publisher: American Physical Society (APS).

[12] Carlström, S., Spanner, M. & Patchkovskii, S. General time-dependent configuration-interaction singles. I. Molecular case. *Physical Review A* **106**, 043104 (2022). Publisher: American Physical Society.

[13] Manolopoulos, D. E. Derivation and reflection properties of a transmission-free absorbing potential. *J. Chem. Phys.* **117**, 9552 (2002). URL http://dx.doi.org/10.1063/1.1517042. Publisher: AIP Publishing.

[14] Nicklass, A., Dolg, M., Stoll, H. & Preuss, H. \emphAb Initio energy-adjusted Pseudopotentials for the Noble Gases Ne Through Xe: Calculation of Atomic Dipole and Quadrupole Polarizabilities. *The Journal of Chemical Physics* **102**, 8942–8952 (1995). URL https://doi.org/10.1063/1.468948.

[15] Tao, L. & Scrinzi, A. Photo-electron momentum spectra from minimal volumes: the time-dependent surface flux method. *New Journal of Physics* **14**, 013021 (2012). URL http://dx.doi.org/10.1088/1367-2630/14/1/013021. Publisher: IOP Publishing.

[16] Zatsarinny, O. BSR: B-spline atomic R-matrix codes. *Computer Physics Communications* **174**, 273–356 (2006). URL http://dx.doi.org/10.1016/j.cpc.2005.10.006. Publisher: Elsevier BV.

[17] Zatsarinny, O. & Bartschat, K. Relativistic B-Spline R-matrix Method for Electron Collisions With Atoms and Ions: Application To Low-Energy Electron Scattering From Cs. *Physical Review A* **77**, 062701 (2008). URL https://doi.org/10.1103/physreva.77.062701.

[18] Fischer, C. F. & Zatsarinny, O. A B-Spline Galerkin Method for the Dirac Equation. *Computer Physics Communications* **180**, 879–886 (2009). URL http://dx.doi.org/10.1016/j.cpc.2008.12.010.

[19] Zatsarinny, O. & Froese Fischer, C. DBSR_hf: A B-spline Dirac–Hartree–Fock program. *Computer Physics Communications* **202**, 287–303 (2016). URL http://dx.doi.org/10.1016/j.cpc.2015.12.023. Publisher: Elsevier BV.

[20] Bartschat, K., Fischer, C. F. & Grum-Grzhimailo, A. N. A Tribute To Oleg Zatsarinny (1953–2021): His Life in Science. *Atoms* **9**, 53 (2021). URL http://dx.doi.org/10.3390/atoms9030053.

[21] Pritchard, B. P., Altarawy, D., Didier, B., Gibson, T. D. & Windus, T. L. New Basis Set Exchange: an Open, Up-To-Date Resource for the Molecular Sciences Community. *Journal of Chemical Information and Modeling* **59**, 4814–4820 (2019). URL http://dx.doi.org/10.1021/acs.jcim.9b00725.

[22] Feller, D. The Role of Databases in Support of Computational Chemistry Calculations. *Journal of Computational Chemistry* **17**, 1571–1586 (1996). URL http://dx.doi.org/10.1002/(SICI)1096-987X(199610)17:13<1571::AID-JCC9>3.0.CO;2-P.

[23] Schuchardt, K. L. *et al.* Basis Set Exchange: a Community Database for Computational Sciences. *Journal of Chemical Information and Modeling* **47**, 1045–1052 (2007). URL http://dx.doi.org/10.1021/ci600510j.

[24] Dunning, T. H. Gaussian Basis Sets for Use in Correlated Molecular Calculations. I. the Atoms Boron Through Neon and Hydrogen. *The Journal of Chemical Physics* **90**, 1007–1023 (1989). URL https://doi.org/10.1063/1.456153.

[25] Jong, W. A. d., Harrison, R. J. & Dixon, D. A. Parallel Douglas–Kroll Energy and Gradients in NWChem: Estimating Scalar Relativistic Effects Using Douglas–Kroll Contracted Basis Sets. *The Journal of Chemical Physics* **114**, 48–53 (2001). URL http://dx.doi.org/10.1063/1.1329891.

[26] Kendall, R. A., Dunning, T. H. & Harrison, R. J. Electron Affinities of the First-Row Atoms Revisited. Systematic Basis Sets and Wave Functions. *The Journal of Chemical Physics* **96**, 6796–6806 (1992). URL http://dx.doi.org/10.1063/1.462569.

[27] Woon, D. E. & Dunning, T. H. Gaussian Basis Sets for Use in Correlated Molecular Calculations. V. Core-Valence Basis Sets for Boron Through Neon. *The Journal of Chemical Physics* **103**, 4572–4585 (1995). URL http://dx.doi.org/10.1063/1.470645.

[28] Fedorov, D. G., Koseki, S., Schmidt, M. W. & Gordon, M. S. Spin-Orbit Coupling in Molecules: Chemistry Beyond the Adiabatic Approximation. *International Reviews in Physical Chemistry* **22**, 551–592 (2003). URL http://dx.doi.org/10.1080/0144235032000101743.

[29] Fedorov, D. G. & Finley, J. P. Spin-Orbit Multireference Multistate Perturbation Theory. *Physical Review A* **64**, 042502 (2001). URL http://dx.doi.org/10.1103/PhysRevA.64.042502.

[30] Zeng, T., Fedorov, D. G. & Klobukowski, M. Multireference Study of Spin-Orbit Coupling in the Hydrides of the 6p-Block Elements Using the Model Core Potential Method. *The Journal of Chemical Physics* **132**, 074102 (2010). URL http://dx.doi.org/10.1063/1.3297887.

[31] Schmidt, M. W. *et al.* General Atomic and Molecular Electronic Structure System. *Journal of Computational Chemistry* **14**, 1347–1363 (1993). URL https://doi.org/10.1002/jcc.540141112.

[32] Gordon, M. S. & Schmidt, M. W. Advances in electronic structure theory. In *Theory and Applications of Computational Chemistry*, Theory and Applications of Computational Chemistry, 1167–1189 (Elsevier, 2005). URL http://dx.doi.org/10.1016/B978-044451719-7/50084-6.

[33] Kramida, A. E. & Nave, G. The Ne II spectrum. *The European Physical Journal D* **39**, 331–350 (2006). URL http://dx.doi.org/10.1140/epjd/e2006-00121-4. Publisher: Springer Science + Business Media.
