# Supplementary material for: Table-top all-attosecond transient absorption spectroscopy
Source: Nat Commun. 2026 Jul 28;17:7572. doi: 10.1038/s41467-026-76019-z (PMC13415544; doi:10.1038/s41467-026-76019-z)
Supplement: Supplementary file 2 — Transparent Peer Review file [file 41467_2026_76019_MOESM2_ESM.pdf]

nature portfolio

Peer Review File

Table-top all-attosecond transient absorption spectroscopy

Corresponding Author: Dr Bernd Schütte

This file contains all reviewer reports in order by version, followed by all author rebuttals in order by version.

Version 0:

Reviewer comments:

Reviewer #1

(Remarks to the Author)
The manuscript by Volkov et al. describes an all-attosecond pump–probe transient absorption spectroscopy experiment on gas-phase atoms using a commercial Ti:sapphire laser system followed by few-cycle pulse compression. Attosecond XUV pulses are generated and propagated over a long distance before being spatially split by split-and-delay multilayer mirrors, enabling pump–probe measurements without significant degradation of the temporal resolution in the nearly collinear configuration.

Although the carrier-envelope phase (CEP) is not stabilized, the pump and probe pulses originate from the same XUV pulse and are therefore intrinsically phase-locked. This experimental strategy enables attosecond temporal resolution in transient absorption spectroscopy without the experimental complexity typically associated with CEP stabilization.

For Xe+, the authors demonstrate clear oscillatory transient absorption signals consistent with coherent hole motion induced by spin–orbit splitting. The agreement between the observed oscillation period and the expected energy splitting provides convincing evidence of attosecond electronic coherence generated by the pump pulse. In contrast, the measurements performed in Ar+ and Ne+ go beyond a demonstration of coherence and provide deeper physical insight into the dynamics. In particular, the phase relationships observed in different spectral regions and their interpretation using theoretical modeling offer a consistent picture based on coherent electron wavepacket motion in ionic states.

The experimental implementation is clear and technically well designed, demonstrating that a standard Ti:sapphire laser platform can be extended to perform attosecond pump–probe spectroscopy in the XUV spectral range. Importantly, the work improves the accessibility of all-attosecond spectroscopy by moving beyond large-scale facility-based implementations toward a laboratory-scale realization.
Overall, I find the manuscript to be clearly presented with a novel experimental setup and interesting results. The combination of technical innovation, experimental robustness, and scientific insight represents a substantial advance in attosecond science. I believe the manuscript will be of broad interest to the ultrafast and attosecond science communities and justifies publication in Nature Communications.

Reviewer #2

(Remarks to the Author)
I have been asked to provide a review of the manuscript entitled “Table-top all-attosecond transient absorption spectroscopy,” submitted by Volkov et al., which describes the realization of an attosecond-pump/attosecond-probe measurement using a table-top high-harmonic generation (HHG) source. The manuscript presents a systematic investigation of electron dynamics in a series of ionized noble gases (Xe, Kr, Ne). I found the manuscript to be clear (for the most part) and well written.

While the results of this technique are clearly articulated, I found parts of the introduction to be a bit confusing. A distinction is made between the term attosecond-pump attosecond-probe spectroscopy (APAPS) and all-attosecond transient absorption spectroscopy (AATAS) which is not clear. For instance, my understanding is that reference 20 also claims to be measuring attosecond transient absorption; X-ray/XUV absorption is routinely performed at synchrotron facilities by scanning the incoming photon energy and recording the yield of photoproducts, which is a direct measurement of the absorption. Describing the present experiment as an ‘absorption’ measurement and the free-electron laser experiment as not is rather confusing (especially for the very large research community who routinely perform x-ray absorption using photoproduct

yield), and possibly inaccurate.

Additionally a substantial fraction of the introduction is devoted to contrasting the table-top source with the free electron laser facility, but the two operate in very different wavelength ranges. The conclusion states that “sufficiently high intensities have already been demonstrated around 100 eV,” but this is still quite far from reaching core-level absorption in biologically relevant samples (which are described as a major motivation in the introduction) and might be difficult to reach the saturation intensity described at the bottom of page 2. In my view, this comparison fails to acknowledge the most exciting advances of the present work and is probably best toned down before publication.

I did have a few more technical questions that would be good to see the Authors address prior to publication:
- In panel b of Figure 2, it looks like the coherent oscillation starts before t=0, is this due to a pre-pulse or is there a different explanation? It seems this behavior does not repeat in the measurement shown in Figure 3.
- At the bottom of page 3, the authors assert that a key factor for AATAS experiments is the stability of the source, and they quote a stability of the NIR pulse energy. Do they have a measure of the shot-to-shot fluctuation of the HHG flux? This seems likely to be the more important factor, not the IR stability (even though they are likely related).
- The authors mention in the final paragraph that noise could be reduced in this measurement using more advanced forms of data analysis. Did the authors attempt to implement any of these analysis techniques? I would be interested to see how these methods improve the already impressive quality of this data.

Overall, provided my concerns above are addressed, I am happy to recommend this work for publication in Nature Communications. While the present work is restricted to atomic systems (presumably because their modeling is relatively straightforward), future work extending this experimental technique to small molecules promises to deliver impactful results relevant to important and outstanding questions on the nature and lifetime of electronic coherence in molecules with multiple nuclear degrees of freedom.

Reviewer #3

(Remarks to the Author)
The authors develop a table-top XUV pump–XUV probe all-attosecond transient absorption spectroscopy (AATAS) technique based on an attosecond high-harmonic generation (HHG) source using a split mirror configuration. Benefiting from improved stability and repetition rate, the work enables the observation of real-time electronic coherent oscillations created by broadband XUV ionization in gas samples. Using this approach, the authors observe coherent hole motion in Xe^+ with a 3.2 fs oscillation period.

The demonstrated AATAS technique provides a more direct measurement of attosecond electronic dynamics with reduced background compared to previously demonstrated ion or electron spectroscopy methods, and it may enable studies in thicker samples. Overall, the manuscript presents an important technical advance, and I recommend publication after the following comments are addressed:

1. Pump fluence comparison with XFEL experiments
In the Table-top AATAS section, the authors discuss the importance and limitations of the pump fluence per laser shot and compare their results with LCLS water AATAS measurements, emphasizing that the transient signal strengths are comparable. However, without quantitative information on the ionization cross section and sample thickness (optical density), it is difficult to conclude that the signal levels are generally comparable to XFEL-based experiments.
It may also be appropriate to note that high-intensity XFEL sources uniquely enable nonlinear X-ray processes and nonlinear probe spectroscopies, such as X-ray stimulated Raman scattering demonstrated in recent work (Li, Kai, et al., Nature 643, 662–668 (2025)). Including such context would provide a more balanced comparison between table-top and XFEL capabilities.

2. Possible wavelength-dependent time-zero shift
Figure 2 presents the AATAS results of coherent electronic motion in Xe^+. Because the employed XUV pulses are spectrally broadband, dispersion effects in the sample may lead to wavelength-dependent group delays, producing a frequency-dependent time-zero shift.
Figure 2b appears to show indications of such wavelength-dependent timing offsets. This effect should either be corrected for or explicitly discussed, as it may influence the interpretation of the phase relationships and the reported out-of-phase oscillations at different photon energies discussed later in the manuscript.

3. Interpretation of phase information in Fig. 3b
The authors state: “Integrated signals across three distinct energy regions are displayed in Fig. 3b, showing that oscillations at these different photon energies are out of phase.”
This conclusion is not fully clear from the current presentation. While Fourier analysis of the AATAS signals reveals oscillation periods, Fig. 3b does not explicitly display phase information. Therefore, it is not straightforward to infer phase differences directly from the figure as shown. The authors should clarify how the phase was extracted or present the phase information explicitly.

4. Interpretation of out-of-phase oscillations
The theoretical calculations are helpful in explaining the wavelength-dependent oscillations. However, the conclusion attributing the out-of-phase behavior to population dynamics may be misleading. After pump ionization, the populations of

the superposition states should remain constant; the observed oscillations arise from phase coherence within the superposition state.
Therefore, the out-of-phase oscillations should instead be attributed to phase differences (coherences) among transitions initiated by the pump pulse. This interpretation appears consistent with the coherence terms listed in Table S1 of the Supporting Information and should be clarified accordingly.

5. Minor issues and typographical corrections
The manuscript is generally well written, and the Supporting Information is clear and well organized. A few minor issues should be corrected:
In the equation describing the two spin–orbit states, the ket–bra notation is used on the left-hand side but not consistently on the right-hand side.
Reference 6 appears to be cited incorrectly.
A careful proofreading for minor typographical errors is recommended.

Version 1:

Reviewer comments:

Reviewer #2

(Remarks to the Author)
The revised manuscript by Volkov et al. entitled “Table-top all-attosecond transient absorption spectroscopy,” has addressed some of my initial comments, and I think the work could be published. It seems the authors changed the order of the references, and then read my comments according to their new ordering. For example, the discussion of ATAS/APAP, the new reference 14 (previous Ref. 20), claims to measure the time-resolved absorption of the sample, which seems to contrast the discussion in the introduction. In my view, this is not an important distinction, but it seems the authors have spent considerable space trying to describe the difference.

I also note that I did not see any changes made to the manuscript in response to either my comments or the comments of the other referee on the wavelength-dependent onset time in the xenon data. There were two speculations in their response to Referee 3, but I would have expected these comments to drive changes to the manuscript, at least enumerating these potential causes of features in the data.

In general, the manuscript is well written, the results are in general encouraging for this technique.

Reviewer #3

(Remarks to the Author)
The authors have satisfactorily addressed my comments, and I recommend publication of the manuscript.

Open Access This Peer Review File is licensed under a Creative Commons Attribution 4.0 International License, which permits use, sharing, adaptation, distribution and reproduction in any medium or format, as long as you give appropriate credit to the original author(s) and the source, provide a link to the Creative Commons license, and indicate if changes were made.
In cases where reviewers are anonymous, credit should be given to 'Anonymous Referee' and the source.
The images or other third party material in this Peer Review File are included in the article's Creative Commons license, unless indicated otherwise in a credit line to the material. If material is not included in the article's Creative Commons license and your intended use is not permitted by statutory regulation or exceeds the permitted use, you will need to obtain permission directly from the copyright holder.
To view a copy of this license, visit https://creativecommons.org/licenses/by/4.0/

We would like to thank the referees for their positive evaluation of our work. In the following, we will provide a point-to-point response to their comments.

Reviewer 2

Comment 1:

A distinction is made between the term attosecond-pump attosecond-probe spectroscopy (APAPS) and all-attosecond transient absorption spectroscopy (AATAS) which is not clear.

Attosecond-pump attosecond-probe spectroscopy (APAPS) is used here as a general term that can involve the observation of electrons, ions, or photons. In contrast, all-attosecond transient absorption spectroscopy (AATAS) refers specifically to a technique in which photon absorption is measured. "Transient absorption" is a commonly accepted way to refer to a pump-probe experiment where the detection occurs in the form of a measurement of pump-laser induced changes to the absorption of the probe laser.

For instance, my understanding is that reference 20 also claims to be measuring attosecond transient absorption;

We agree with the referee that Ref. 20 reports an attosecond transient absorption (ATAS) experiment. However, in that work only the XUV probe pulse had attosecond duration, while the NIR pump pulse was in the femtosecond range. To distinguish our approach from the results of Ref. 20, we refer to our technique as *all*-attosecond transient absorption spectroscopy (AATAS), in which both the pump and probe steps use attosecond pulses.

X-ray/XUV absorption is routinely performed at synchrotron facilities by scanning the incoming photon energy and recording the yield of photoproducts, which is a direct measurement of the absorption. Describing the present experiment as an 'absorption' measurement and the free-electron laser experiment as not is rather confusing (especially for the very large research community who routinely perform x-ray absorption using photoproduct yield), and possibly inaccurate.

There is a conceptual difference between our experiment and the experiments described by the referee. When the photon energy is scanned and photoproducts are recorded, only absorption processes that produce photoions or photoelectrons can be observed. Such experiments cannot detect photon absorption that leads to bound-to-bound transitions. In contrast, the results presented in this manuscript rely primarily on bound-to-bound transitions.

Comment 2:

Additionally a substantial fraction of the introduction is devoted to contrasting the table-top source with the free electron laser facility, but the two operate in very different wavelength ranges. The conclusion states that "sufficiently high intensities have already been demonstrated around 100 eV," but this is still quite far from reaching core-level absorption in biologically relevant samples (which are described as a major motivation in the introduction) and might be difficult to reach the saturation intensity described at the bottom of page 2. In my view, this comparison fails to acknowledge the most exciting advances of the present work and is probably best toned down before publication.

The statement referred to by the referee ("sufficiently high intensities have already been demonstrated around 100 eV") is part of a broader outlook and does not specifically address biologically relevant molecules. While discussing specific experiments is beyond the scope of this work, we note that the absorption edges of several elements, including Si, lie in the 100 eV region. While we may not be able to reach the saturation intensity at 100 eV, we still expect to obtain a good SNR due to the low noise in our experiments.

Comment 3:

In panel b of Figure 2, it looks like the coherent oscillation starts before t=0, is this due to a pre-pulse or is there a different explanation? It seems this behavior does not repeat in the measurement shown in Figure 3.

As shown in Fig. 2**c**, the maximum of the signal is observed at time zero. Due to the finite temporal resolution and the comparable large noise, some signal appears at negative time delays. As shown in Fig. 1**c**, the estimated pulse structure includes a pre- and a post-pulse. These effects are negligible in Fig. 3, since the oscillation periods of the coherent wavepacket motion are substantially longer for Ar and Ne. We also refer to our response to comment 2 made by referee 3.

Comment 4:

At the bottom of page 3, the authors assert that a key factor for AATAS experiments is the stability of the source, and they quote a stability of the NIR pulse energy. Do they have a measure of the shot-to-shot fluctuation of the HHG flux? This seems likely to be the more important factor, not the IR stability (even though they are likely related).

In Ref. 27 we have measured the single-shot XUV pulse energy fluctuations as 0.7 % over the time span of five minutes. We have included this information in the SI.

Comment 5:

The authors mention in the final paragraph that noise could be reduced in this measurement using more advanced forms of data analysis. Did the authors attempt to implement any of these analysis techniques? I would be interested to see how these methods improve the already impressive quality of this data.

We are currently testing these techniques in molecules where we have performed a number of measurements. Preliminary results suggest that the SNR can be further improved by a factor of 2-5.

Reviewer 3

Comment 1:

Pump fluence comparison with XFEL experiments

In the Table-top AATAS section, the authors discuss the importance and limitations of the pump fluence per laser shot and compare their results with LCLS water AATAS measurements, emphasizing that the transient signal strengths are comparable. However, without quantitative information on the ionization cross section and sample thickness (optical density), it is difficult to conclude that the signal levels are generally comparable to XFEL-based experiments.

It may also be appropriate to note that high-intensity XFEL sources uniquely enable nonlinear X-ray processes and nonlinear probe spectroscopies, such as X-ray stimulated Raman scattering demonstrated in recent work (Li, Kai, et al., Nature 643, 662–668 (2025)). Including such context would provide a more balanced comparison between table-top and XFEL capabilities.

The higher cross sections in the XUV region when compared to the X-ray regime are an important reason why we can achieve similar signal levels with our table-top source when compared to the result reported from LCLS. We provide cross sections in our manuscript (using σ_{Ar}=34.75 Mb and σ_{Xe} =21.53 Mb at 24 eV), and the gas density in our experiment was adjusted to achieve approximately 50 % static absorption. For instance, the pressure in the Ar experiment was 85 mbar. We cannot make a more general comparison to XFEL experiments, since to the best of our knowledge only one AATAS experiment has been reported so far.

There is no doubt that certain experiments can only been done at X-ray free-electron lasers including the work on X-ray stimulated Raman scattering. However, a comprehensive discussion of such techniques is beyond the scope of our manuscript, which focuses on all-attosecond transient absorption spectroscopy.

Comment 2:

Possible wavelength-dependent time-zero shift

Figure 2 presents the AATAS results of coherent electronic motion in Xe^+. Because the employed XUV pulses are spectrally broadband, dispersion effects in the sample may lead to wavelength-dependent group delays, producing a frequency-dependent time-zero shift.

Figure 2b appears to show indications of such wavelength-dependent timing offsets. This effect should either be corrected for or explicitly discussed, as it may influence the interpretation of the phase relationships and the reported out-of-phase oscillations at different photon energies discussed later in the manuscript.

We have estimated that the wavelength-dependent group delays induced by neutral Xe are negligible. While dispersion increases near Xe^+ resonances, absorption lines with comparable oscillator strengths are expected to show a similar behavior.

In Fig. 2b the line near 16 eV seems to appear earlier than the lines in the 17-18 eV region. This apparent difference may arise from two effects: (i) The noise at 16 eV is comparably high. (ii) The line near 16 eV reaches a maximum at time zero due to coherent wave-packet motion, whereas the lines between 17 and 18 eV lie on the rising slope of the absorption signal at time zero.

Most importantly, the out-of-phase behavior in our manuscript is discussed only for Ar and Ne, where the uncertainties of the relative phases are much smaller due to the higher SNR and the longer oscillation periods. As a result, the point raised by the referee does not affect the conclusions in our paper.

Comment 3:

Interpretation of phase information in Fig. 3b

The authors state: “Integrated signals across three distinct energy regions are displayed in Fig. 3b, showing that oscillations at these different photon energies are out of phase.”

This conclusion is not fully clear from the current presentation. While Fourier analysis of the AATAS signals reveals oscillation periods, Fig. 3b does not explicitly display phase information. Therefore, it is not straightforward to infer phase differences directly from the figure as shown. The authors should clarify how the phase was extracted or present the phase information explicitly.

We have made a mistake in the reference, which we have now corrected. This statement refers to Fig. 3**c** (not Fig. 3**b**), where lineouts taken at different photon energies show a clear out-of-phase behavior.

Comment 4:

Interpretation of out-of-phase oscillations

The theoretical calculations are helpful in explaining the wavelength-dependent oscillations. However, the conclusion attributing the out-of-phase behavior to population dynamics may be misleading. After pump ionization, the populations of the superposition states should remain constant; the observed oscillations arise from phase coherence within the superposition state.

Therefore, the out-of-phase oscillations should instead be attributed to phase differences (coherences) among transitions initiated by the pump pulse. This interpretation appears consistent with the coherence terms listed in Table S1 of the Supporting Information and should be clarified accordingly.

We agree with the referee that the populations of the j=1/2 and the j=3/2 states are time-independent. However, the p_0 orbital that is referred to does not correspond to either the hole in the j=1/2 state, nor the hole in the j=3/2 state. Rather, the holes in the j=1/2 state and the 3/2 state correspond to different linear combinations of the p_0 orbital and the p_1 orbital. Accordingly, the superposition of the j=1/2 and j=3/2 oscillates back and forth between a configuration that is close to a p_0 hole and one that is closer to a p_1 hole.

To avoid potential misunderstandings, we have modified the corresponding statements and removed the term “population”.

“… Upon ionization, a partially coherent wavepacket is created in the ion. The initial shape of the hole wavefunction resembles a $\mathbf{p_0}$ orbital. Subsequently, the wavepacket starts to evolve and oscillates with the characteristic time period given by the spin–orbit splitting. …”

“Transfer of an electron from an occupied 2s orbital to an unoccupied p_0 orbital is maximized when the hole has predominantly p_0 character. Given that the initial shape of the hole wave function resembles a p_0 orbital, the oscillation around 26.85 eV therefore starts with maximum absorption near the time overlap between the pump and the probe pulses. Conversely, excitation to an unoccupied 3s orbital is maximized when the hole is localized on the doughnut (p_1). For this reason, the absorption at 27.75 eV starts near a minimum and gains in strength when the hole acquires p_1 character.”

Comment 5:

The manuscript is generally well written, and the Supporting Information is clear and well organized. A few minor issues should be corrected: In the equation describing the two spin–orbit states, the ket–bra notation is used on the left-hand side but not consistently on the right-hand side. Reference 6 appears to be cited incorrectly.

A careful proofreading for minor typographical errors is recommended.

We have corrected these points and we have carefully proofread the manuscript and the SI. We note that we have double checked Ref. 6 in both the manuscript and the SI, and we have not found a mistake.

On behalf of all co-authors,

Bernd Schütte (Max Born Institute, Berlin)

We would like to thank the referees for their positive evaluation of our work. In the following, we will provide a response to the remaining comment by referee 2.

Reviewer 2

Comment:

I also note that I did not see any changes made to the manuscript in response to either my comments or the comments of the other referee on the wavelength-dependent onset time in the xenon data. There were two speculations in their response to Referee 3, but I would have expected these comments to drive changes to the manuscript, at least enumerating these potential causes of features in the data.

We have added the following statement in the caption of Fig. 2:

"The small signal between -1 fs and 0 fs is attributed to overlap effects between the pump and probe pulses."

On behalf of all co-authors,

Bernd Schütte (Max Born Institute, Berlin)
